# Granuloma Mimicking Local Recurrence on PET/CT after Liver Resection of Colorectal Liver Metastasis: A Case Report

**DOI:** 10.7759/cureus.717

**Published:** 2016-08-01

**Authors:** Eirini V Pantiora, Elissaios A Kontis, Vasiliki Michalaki, Elias Primetis, Antonios Vezakis, Andreas Polydorou, Georgios P Fragulidis

**Affiliations:** 1 2nd Department of Surgery, ARETAIEIO Hospital, National and Kapodistrian University of Athens School of Medicine; 2 Department of Oncology, ARETAIEIO Hospital, National and Kapodistrian University of Athens School of Medicine; 3 1st Department of Radiology, ARETAIEIO Hospital, National and Kapodistrian University of Athens School of Medicine

**Keywords:** fdg-pet, PET/CT, granuloma, liver metastases, colorectal cancer, metastasectomy

## Abstract

Positron emission tomography–computed tomography (PET/CT) improves the diagnostic interpretation of fluorine-18 fluorodeoxyglucose (18F-FDG ) PET and CT in oncologic patients and has an impact on both diagnostic and therapeutic aspects of patient management. However, false positive findings from the PET/CT imaging should be taken into consideration as they mislead physicians into improper therapeutic actions. We present a 48-year-old female patient with a history of left colectomy for colorectal cancer and subsequent liver metastasectomy. After one year of follow-up, she presented with a highly suspicious lesion in the liver, which was confirmed on PET/CT as a metastatic liver tumor. Consequently, the patient underwent surgical excision of the tumor, and the definitive histological diagnosis showed a granulomatous tissue with giant cells and foreign body tissue reaction. Based on this report, we briefly review the dangerous pitfalls from radiological and PET/CT imaging concerning the preoperative diagnostic workup examination, as they may significantly alter the treatment plan in oncologic patients.

## Introduction

Staging and follow-up of oncologic patients rely widely on radiologic imaging modalities' accuracy that can in turn directly influence therapeutic decisions. ^18^FDG - PET/CT scan benefits from the combination of functional and structural information providing a highly superior diagnostic accuracy and has been widely used in the detection of distant metastases [1]. As the liver is one of the commonest sites of metastasis, in particular for the tumors arising from the colon and rectum, liver imaging presents a common challenge in oncological evaluation by PET/CT.

The practice of PET/CT in patients with colorectal cancer presented with hepatic metastases has been shown to improve therapeutic planning by detecting intrahepatic and extrahepatic sites of the disease. Liver metastases from colorectal cancer represent approximately 50% of the recurrences, and surgical resection is the only potentially curative therapy in these patients. In contrary, PET/CT may eliminate ineffective surgery by demonstrating additionally extrahepatic metastases with the inoperable disease in 11-32% of patients. This approach alters the therapeutic management to a more systemic approach with chemotherapy [2].

Despite PET/CT being a highly accurate imaging method, it is still susceptible to artifacts and pitfalls that compromise its efficiency [3]. Herein we present the case of a 48-year-old female patient with metastatic colorectal cancer, who was subjected to an avertable hepatic tumor resection following misleading findings in PET/CT scan, throughout her follow up. She underwent surgical excision of the suspicious tumor, which proved to be granulomatous tissue, secondary to inflammation with no metastases in the pathology report.

## Case presentation

**Figure 1:**
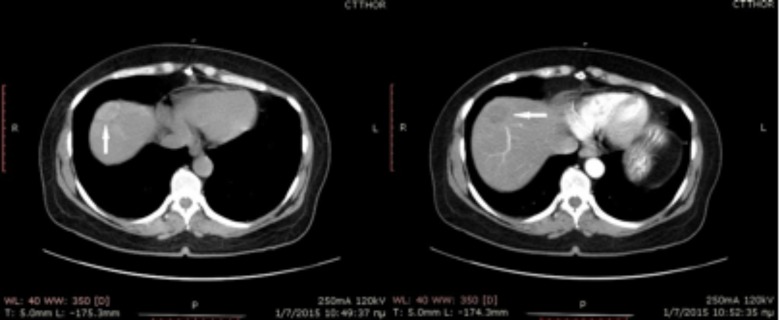
Axial contrast-enhanced CT shows a hypodense lesion at metastasectomy site. The lesion enhances after contrast administration.

A 48-year-old female patient with a past medical history of left colectomy and metastasectomy in liver segments VII and III, presented after one year of follow-up with a suspicious lesion in the resection margin of segment VII on a CT scan as can be seen in Figure 1.

**Figure 2:**
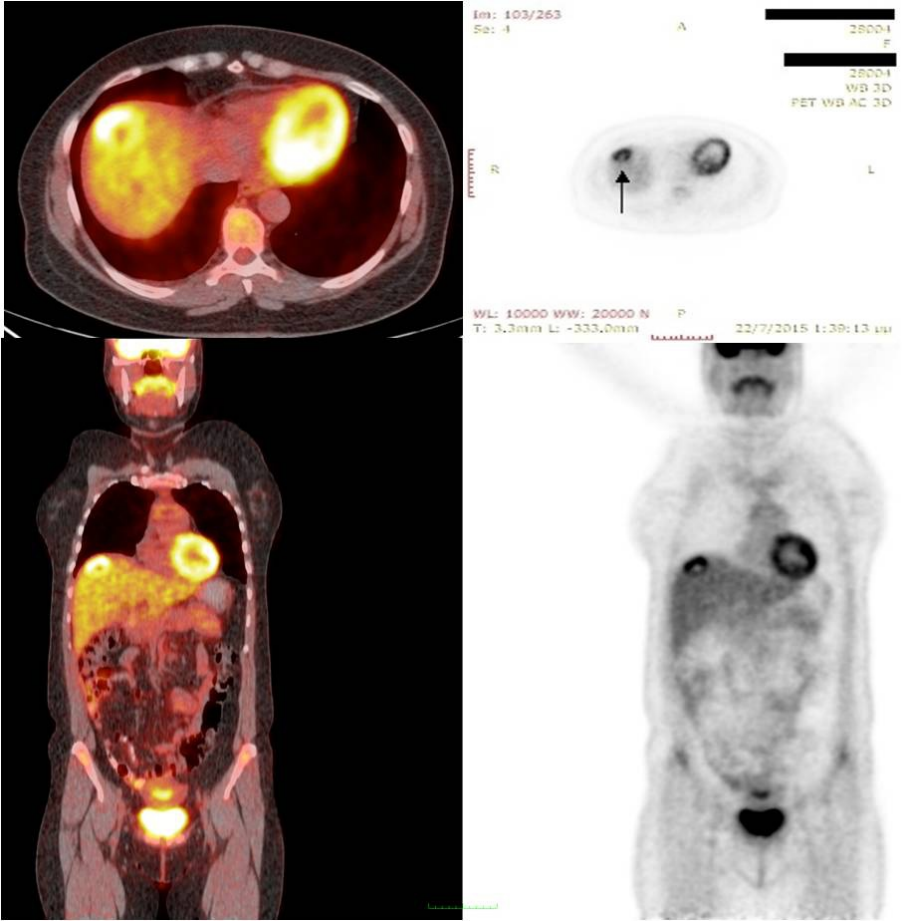
Eighteen PET/CT scans reveal that the lesion is hypermetabolic with a standardized uptake value (SUVmax) of 8.

The PET/CT confirmed the presence of a tumor with increased SUV (Standardized Uptake Value) of eight, as can be seen in Figure 2.

**Figure 3:**
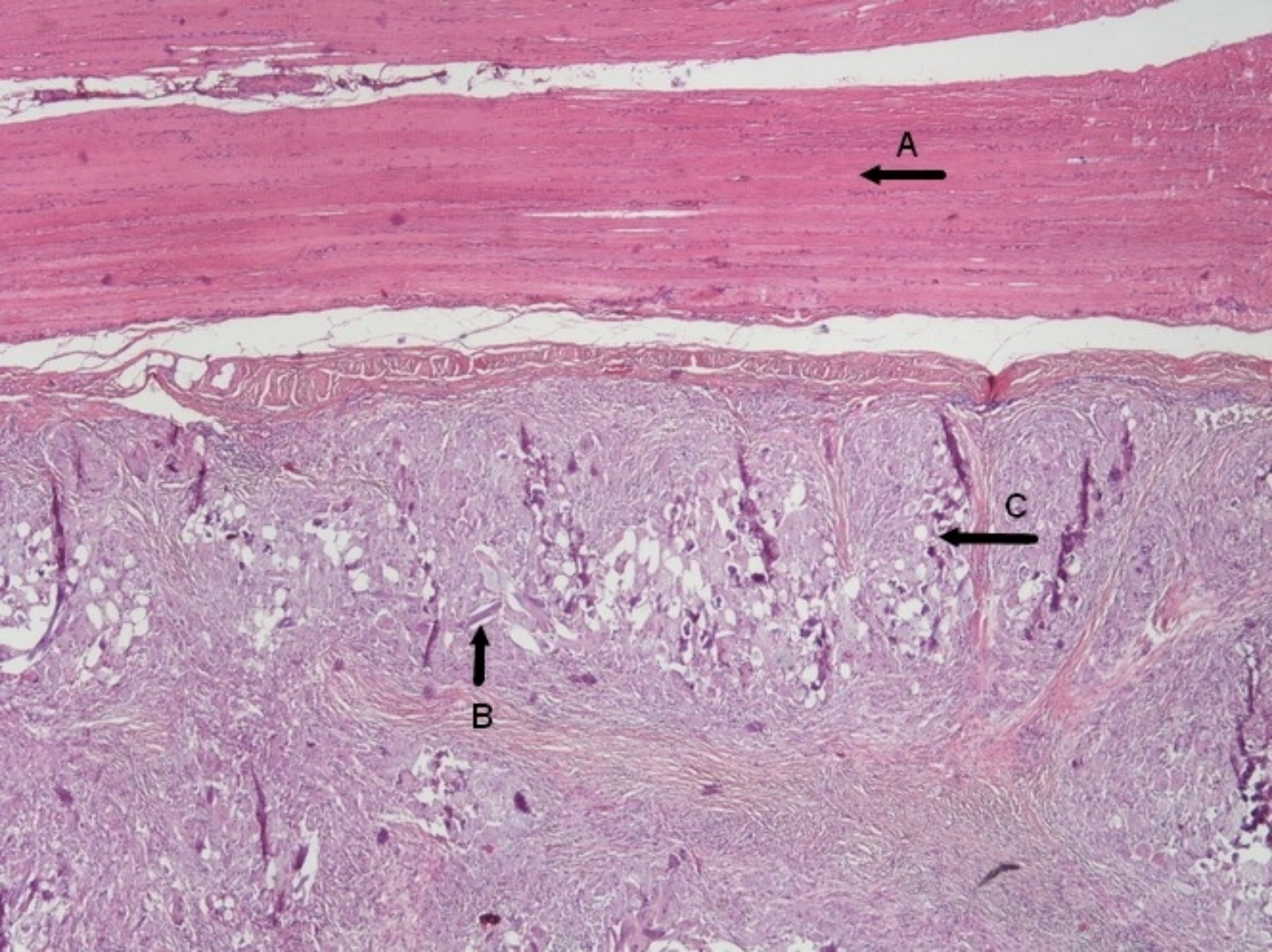
Low power histology section showing the diaphragm muscle (A), foreign body remnants (B) and giant cells (C).

**Figure 4:**
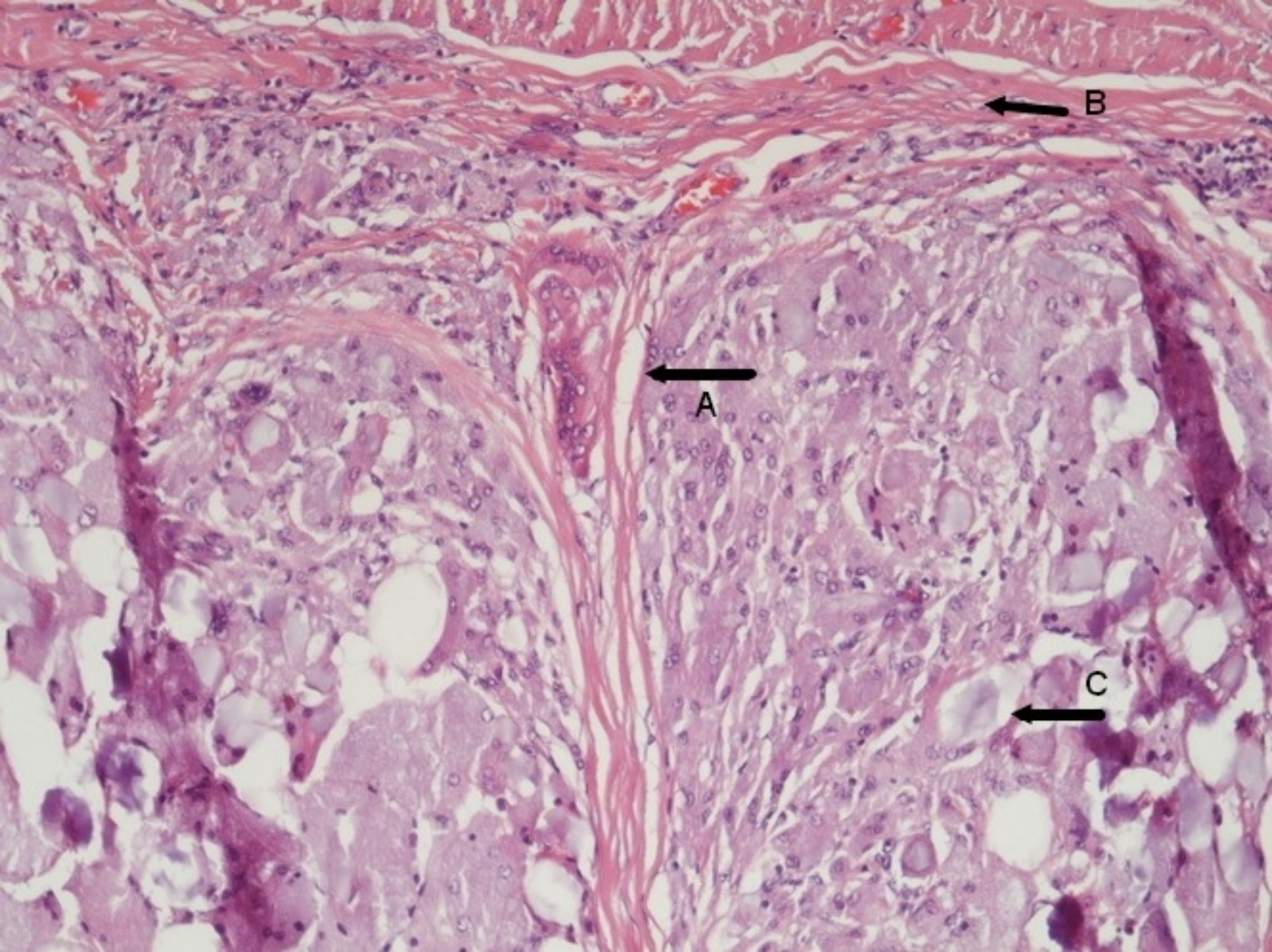
Low power histology section showing giant cells (A), foreign body remnants (B), fibrous tissue (C) and hepatic tissue with adjacent foreign body reaction.

The serum tumor markers were within normal range. With a provisional diagnosis of local recurrence, the patient was scheduled for an exploratory laparotomy and liver metastasectomy. An intraoperative ultrasound was performed, which detected a lesion of approximately 4 cm at the site of previous resection adherent with the diaphragm. An en bloc resection of the tumor and part of the diaphragm followed. A frozen section of the resected liver specimen was negative for malignancy. The histology revealed granulomatous tissue with giant cells and foreign body tissue reaction as presented in Figure 3 and Figure 4.

The patient had an uneventful postoperative course and was discharged home on the fifth postoperative day.

## Discussion

PET/CT has been established as an indispensable imaging tool in oncologic patients. It is widely used for diagnosis, staging, and monitoring of several malignancies, and entails an excellent supplemental testing for clarification of suspicious findings detected in a CT scan [1]. Interpretation of a PET/CT scan, however, requires vigilance and experience to identify false positive findings. The common benign pathology may mimic malignancy in these scans, which may lead to an ill-suited therapeutic strategy with possible negative repercussions.

Metallic implants or presence of contrast medium is a fairly common factor that causes false positive findings in a PET/CT image. Also, patient breathing during the procedure might lead to misalignment of the images, and inaccurate localization of a lesion, i.e. a liver lesion might be interpreted as a lung nodule due to diaphragm movement. The above artifacts can be eliminated through technical practices and careful instructions towards the patient regarding his breathing movements [4]. Nevertheless, apart from the general technology-specific pitfalls, there are imaging issues that are specifically related to the liver which is one of the commonest sites of metastasis arising from the gastrointestinal tract, lung, pancreas and other primary tumors.

Because the diagnosis of hepatic metastasis often alters the treatment plan considerably, liver imaging presents a common challenge in oncological evaluation by PET/CT. It is important to take into consideration that ^18^FDG (18-fluoro-2-deoxyglucose) uptake is not specific to malignancy. ^18^FDG is a glucose analog that allows evaluation of glucose metabolism and is the most commonly used PET tracer that is transported into cells by glucose transporters. Cancer cells have been shown to indicate an increased uptake of glucose and glycolytic enzymes, and therefore preferential uptake of FDG. Yet, ^18^FDG is well known to accumulate in inflammatory cells such as lymphocytes, neutrophils and macrophages in various inflammatory conditions due to elevated glucose requirements. The increased uptake of FDG in such lesions mimics the imaging characteristics of malignant tissue leading to false positive results [5].

Therefore, correlation with clinical setting and other imaging findings is essential for the correct diagnosis. For instance, the presence of solitary uptake in the liver is non-specific of metastases in PET/CT imaging. On the other hand, multiple sites of focally increased ^18^FDG concentration, in the background of relatively lower, normal hepatocyte-uptake are regarded as the hallmark of metastatic liver involvement from a known primary tumor. Solitary uptake can be due to infection or other inflammatory conditions like histiocytosis, abscesses, and granulomatous tissue. The inflammatory process can produce granulomatous tissue, i.e. sarcoidosis or as a reaction to foreign bodies like surgical adhesives, hemostatic agents or sutures [6,7]. Similarly, inflammation related to therapeutic procedures such as surgery often produce a foreign body reaction leading to increased uptake on PET/CT scan following surgery or biopsy [8]. Foreign body granulomas have also been reported to masquerade as liver metastases in radiologic imaging [9]. This phenomenon is particularly common after lung resections, where adhesives are used to prevent air leakage but have also been reported in general surgical cases after the use of hemostatic agents [7].

Taking into consideration the impact of PET/CT findings on therapeutic decisions regarding oncologic patients, the occurrence of false positive results is not negligible, as PET/CT can direct patient management by targeting surgical resections of liver metastases. Nevertheless, although knowing these pitfalls, the physician should be assessed the outcome of undertreating a malignancy and the mortality and morbidity risks of a surgical procedure. Our patient had already been subjected to metastasis excision while both CT and PET/CT scans confirmed the presence of a highly suspicious lesion and no evidence of widespread disease. SUV (Standardized Uptake Value) on PET/CT revealed an increased uptake of eight, exceptionally higher than the cutoff level between benign and malignant lesion or nodule; the cutoff being approximately 3.5 for metastases > 1 cm, and could vary across between several tumors [10]. Hence, in our case, exploratory surgery seemed the only reasonable option. 

## Conclusions

Abnormal liver uptake in PET/CT imaging is a common finding and interpretation of radiologic images in oncologic patients. It should be approached with caution and awareness of pitfalls, especially in the postoperative setting. Correlation with an appropriate clinical background, and findings from other imaging modalities and tumor marker values, are the keys to decision making in achieving a correct diagnosis. In doubtful cases, other diagnostic methods should be employed whenever possible, i.e., percutaneous or endoscopic biopsy. 
